# The Dynamics of Radio-Cesium in Soils and Mechanism of Cesium Uptake Into Higher Plants: Newly Elucidated Mechanism of Cesium Uptake Into Rice Plants

**DOI:** 10.3389/fpls.2020.00528

**Published:** 2020-05-13

**Authors:** Hiroki Rai, Miku Kawabata

**Affiliations:** Department of Biological Production, Faculty of Bioresource Sciences, Akita Prefectural University, Akita, Japan

**Keywords:** cesium, potassium transporters, HAK, KIR channels, VIC channels, radio-cesium, rice, Fukushima

## Abstract

Soil radio-cesium (Cs) contamination caused by nuclear accidents is a major public concern. In this review, we presented the behavior of radio-Cs in soils, the relationship between Cs^+^ and potassium (K) ion uptake from soils, and the Cs^+^ uptake model proposed previously. Finally, we introduced the newly elucidated mechanism of Cs^+^ uptake in rice plants and compared it with the previously proposed Cs^+^ uptake model. Cs is a trace element in soil. It is toxic to plants when absorbed at high concentrations, although this rarely occurs under normal environmental conditions. Nevertheless, radio-Cs released during nuclear weapon tests or nuclear power plant accidents is absorbed by plants, thus entering the food chain. As Cs^+^ strongly binds to the frayed edge sites of illitic clays in soil, it is hardly moved by the infiltration of rainwater. However, plants have a strong ability for inorganic ions uptake, causing re-diffusion of radio-Cs^+^ into ecosystems and radioactive contamination of food. It is hypothesized that Cs^+^ is absorbed by plants through the same mechanism implemented in K^+^ uptake. However, the dynamics of the two elements do not always coincide in their transition from soil to plants and inside the plants. A previously proposed model of Cs uptake by higher plants stated that Cs^+^ is absorbed through high affinity potassium (HAK) family of transporters and voltage-insensitive cation (VIC) channels. A knockout line of a HAK transporter gene (*oshak1*) in rice revealed that the HAK transporter OsHAK1 is the main route of Cs^+^ influx into rice plants, especially in low-potassium conditions. The K^+^ uptake rates did not differ greatly between the *oshak1* and wildtype. On the surface of rice roots, potassium-transport systems other than OsHAK1 make little or no contribution to Cs^+^ uptake. It is almost certain that OsAKT1 does not mediate the Cs uptake. Under normal soil conditions, 80–90% of Cs uptake into the roots is mediated by OsHAK1 and the rest by VIC channels. Except for the difference between the contribution ratio of HAK and VIC channels in Cs uptake, these results are consistent with the conventional model.

## Introduction

Cesium (Cs) is a Group I alkali metal along with sodium (Na) and potassium (K). Its chemical properties are very similar to those of K, which is one of the macronutrient elements of plants. Generally, the solubility of K and Cs salts is very high, and both are always present as monovalent ions in the natural environment. The concentration of K^+^ is in the range from 100 to 150 mM in the cytoplasm of plant cells, where it regulates the osmotic pressure of the cells. The controlled osmotic fields are essential for enzymatic reactions and maintaining the structure of nucleic acids. Although Cs^+^ has physical properties similar to K^+^, Cs^+^ does not provide the same as K^+^ in vital cell activities. As Cs concentration of the cells increases, the cytotoxicity increases due to the decrease in or inhibition of the enzyme activity (White and Broadley, 2000; Hampton et al., 2004; Maathuis, 2009).

The concentration of stable Cs isotope (^133^Cs) in the environment is generally low (up to 25 μg/g soil) and not harmful to plants or human health. However, Cs uptake by plants may become a public concern when radio-Cs (^134^Cs, ^137^Cs) is released by nuclear weapons tests or nuclear power plant accidents. Plants can absorb the radio-Cs in soil and incorporate it into the food chain, where it may cause internal exposure to β and γ radiation during its radioactive decay (White and Broadley, 2000).

The research on the absorption of Cs^+^ from soil by plants has been intensive over the past several decades. Cs^+^ and K^+^ compete during uptake by plants and a Cs^+^ uptake model has been proposed based on the analysis of K^+^ transporters. After the nuclear accident caused by the Great East Japan Earthquake in 2011, research on Cs^+^ absorption in rice has been advanced in Japan. This helped to elucidate the mechanism of Cs^+^ absorption by rice roots.

In this review, we introduce the outline of Cs^+^ dynamics in soil and the results of research on the absorption mechanism in plants. Then, we compare the conventional Cs^+^ absorption model of plants with the newly elucidated mechanism of Cs^+^ uptake into rice.

## Cesium and Radio-Cesium Existence in the Environment

The concentration of Cs (^133^Cs) in soil is low. Although Cs is toxic to plants when absorbed in high concentration as its intracellular concentration becomes high, such a phenomenon rarely occurs under normal environmental conditions. The toxicity of Cs to living organisms is not from the stable isotope (^133^Cs), but from the artificially produced radioactive Cs (^134^Cs, ^137^Cs). Atmospheric nuclear tests have already been prohibited, but the commercial nuclear power reactors account for 10% of electricity produced in the world.

We have experienced widespread radionuclide contaminations caused by the nuclear power plant accidents in the Chernobyl Nuclear Power Plant, Ukraine, in 1986 and in the Fukushima Daiichi Nuclear Power Plant, Japan, in 2011. Radio-Cs was a common problem in both nuclear accidents.

In the Chernobyl accident, the containment vessel exploded, exposing the reactor core to the atmosphere and releasing numerous radionuclides such as radioactive xenon (^133^Xe), iodine (^131^I), Cs (^134,137^Cs), strontium (^90^Sr), zirconium (^95^Zr), ruthenium (^103,106^Ru), and plutonium (^239^Pu) (International Atomic Energy Agency [IAEA], 2003–2005; Imanaka, 2016). Among these nuclides, radio-Cs contaminated a large area and became a major source of radioactive contamination of the food chain (Fesenko et al., 2007).

In the Fukushima disaster, the containment vessel itself was not severely damaged, but ^133^X, ^131^I, and ^134,137^Cs leaked from the containment vessel and were released into the atmosphere by venting or by hydrogen explosion of the building. ^133^Xe, which is a gas, and ^131^I and ^134,137^Cs, both with low boiling points, were released in large amounts and widely dispersed. Of these three elements, ^133^Xe diffused into the atmosphere, and ^131^I was not detected in a few months after the accident because of its short half-life (8.04 days). However, the half-life of radio-Cs is long (2.06 years for ^134^Cs; 30.17 years for ^137^Cs), and it tends to strongly adsorb to the soil, thus polluting the fallout area for a long time (Chino et al., 2011; Beresford et al., 2016; Imanaka, 2016).

**FIGURE 1:**
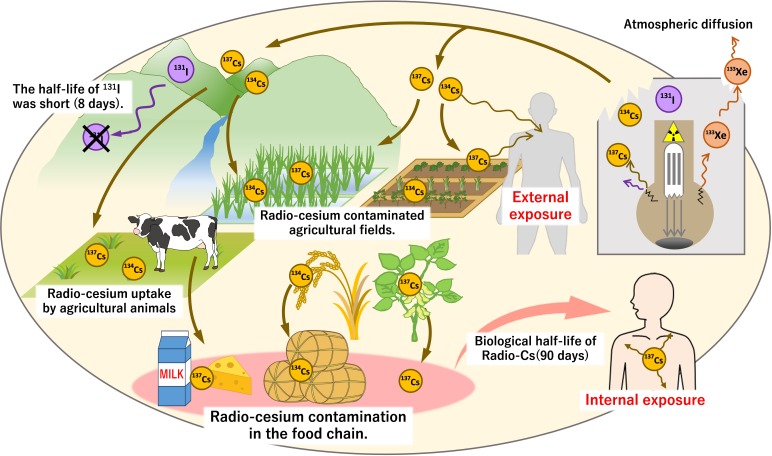
Diffusion of radio-Cs and contamination of soil and food due to the Fukushima nuclear plant accident. At the time of the nuclear power plant accident, gaseous ^133^Xe as well as ^131^I, ^134^Cs, and ^137^Cs with low boiling point easily leaked. These nuclides were released and spread widely when a hydrogen explosion damaged the plant building. ^133^Xe diffused quickly into the atmosphere, and ^131^I could not be detected within a few months due to its short half-life. In contrast, the half-lives of ^134,137^Cs are long, allowing its persistence in the environment for long periods. The decay of ^134,137^Cs caused external exposure to emitted γ-rays. Internal exposure also occurred through the ingestion of contaminated food. Absorption of ^134,137^Cs by plants (agricultural crops) allowed its entry into the food chain resulting in the contamination of grains, vegetables, dairy products, and many more food sources. Due to the long biological half-life of Cs in humans (approximately 90 days), it is necessary to monitor these isotopes in soil, plants, and foods.

There are two problems related to radio-Cs pollution of the environment. One is elicited through external exposure, in which β and γ rays are emitted by the decay of radio-Cs, so air dose increases, exposing people to these harmful rays. The other problem is related to internal exposure generated through absorption of radio-Cs by crops and its transfer into the food chain, contaminating food and posing health problems (Fesenko et al., 2007; Beresford et al., 2016). The rate of radio-Cs absorption from ingested contaminated food is extremely high; the transfer factor is 65–90% (Henrichs et al., 1989; Beresford et al., 2000). In mammals, radio-Cs, similar to K, is taken up into the blood and transferred into tissues such as muscles, but its fecal and urinary excretion is low. In the body, radio-Cs has a considerably long half-life (45–200 days, with an average of 90 days), exposing the internal organs to radiation from radio-Cs (Henrichs et al., 1989). In addition, herbivores absorb radio-Cs from pasture fields, which results in contamination of dairy products and meat. In Chernobyl, the consumption of foods containing radio-Cs was the major source of public exposure via milk and other animal products (Beresford et al., 2000; Fesenko et al., 2007; Fesenko et al., 2015; Figure 1). In Fukushima, the provisional guideline levels for radio-Cs were specified immediately after the accident and all agricultural products were measured for radioactivity before shipment. The products contaminated with excess radiation have not been circulated (Nihei, 2013). Internal exposure by foods originates from the absorption of radio-Cs by plants, so it is important to know the dynamics of Cs from soils to plants.

## Dynamics of Cesium and Radio-Cs in the Environment

Cesium has a strong ionization tendency, and its salts generally have high solubility. The radio-Cs released from nuclear power plants is deposited from the atmosphere onto the ground. The particles containing radio-Cs fall on the soil surface in crop fields or on the leaves and bark of forest trees, and eventually enter the organic matter layer with rainfall. Ultimately, as the organic matter decomposes, radio-Cs migrates into the A layer containing clay minerals, where it behaves differently from K^+^ and Na^+^ (Takahashi et al., 2019).

**FIGURE 2:**
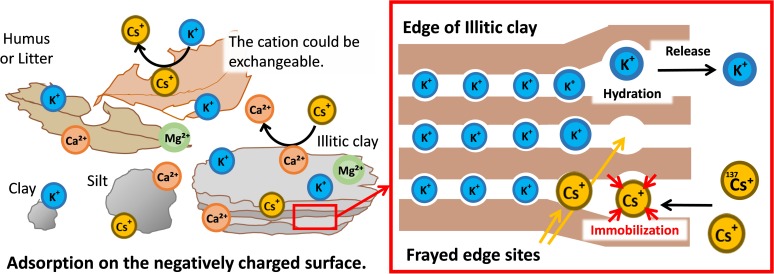
Dynamics of radio-Cs in soil and the quantitative relationship between potassium and Cs. In soil, radio Cs^+^ is adsorbed through the negative charges of organic substances such as humus or clay similarly to other cations such as K^+^ and Ca^2^. In addition, Cs^+^ is strongly immobilized (captured) on the mica’s frayed edge sites over time. As a result, radio Cs^+^ is vertically immobile and mostly accumulates on the top soil.

Clay and humus contained in soil are negatively charged and have cation exchangeable sites that commonly adsorb cations such as Cs^+^ and K^+^ (Figure 2, left panel). Furthermore, there are sites in the soil that specifically adsorb Cs^+^ (Cremers et al., 1988). Clay minerals in the soil have a layered structure of aluminosilicates, with the layers whose surfaces are negatively charged and held together by cations such as K^+^ and Mg^2+^. As soil weathers, aluminosilicates are precipitated, the ions between the layers are gradually released from the outer edge, and the layers slightly open at the edge of the clay mineral. In illitic clays, this edge is named frayed edge site, and it shows a specific adsorption affinity for Cs^+^ whose ionic radius is large and hydration radius is small. The adsorption of Cs^+^ by frayed edge sites is more than 1000-fold greater than that for K^+^, and it is almost irreversible (Yamaguchi, 2014; Figure 2, right panel).

However, soils lacking illitic clays have much higher Cs availability for plant uptake. Thus, Radio-Cs Interception Potential (RIP) was proposed as the quantitative measure of radio-Cs retention in soils (Cremers et al., 1988). In typical clay soils containing illitic clays, the number of this frayed edge sites is much higher than that required for the immobilization of radio-Cs (Cremers et al., 1988; Vandebroek et al., 2012). It is assumed that most of the stable isotope Cs^+^ is trapped by this frayed edge sites. Newly supplied Cs^+^, such as radio-Cs, is hardly moved in the soil because it is captured by these sites and thereby immobilized. In the absence of human disturbance, most of the radioactive Cs^+^ from the nuclear accidents has been concentrated in the top several centimeters of the soil (Almgren and Isaksson, 2006; Takahashi et al., 2019).

The concentration of stable isotope Cs (^133^Cs) in a typical soil is 2.5 mg kg^–1^ and the concentration of Cs^+^ in soil solution is lower than 10 μg L^–1^ (75 nM) (Tsukada et al., 2002; Rai et al., 2017). The soil concentration of radio-Cs is considerably lower than the concentration of ^133^Cs.

In Japan, a reference value was set for protection during the continuous consumption of radioactive contaminated food after the Fukushima nuclear accident. The concentration of radio-Cs in soil where radioactive contamination of crops would exceed the reference value could be estimated from the transfer factor and the reference value of crops (100 Bq kg^–1^ in rice). In the case of rice, the transfer factor for brown rice from the soil is from 0.004 to 0.065 (Kondo et al., 2015). At 0.065 as transfer factor, the radio-Cs concentration in the soil is estimated to 1540 Bq kg^–1^ soil. The concentration of radio-Cs in soils where agriculture could resume is expected to be less than a several thousand Bq kg^–1^ soil.

In soils contaminated with ^134^Cs or ^137^Cs at 10,000 Bq kg^–1^ soil, the weight of Cs can be calculated using the following formula.

$$\begin{array}{l} 10,000\;{\text{Bq;}}^{134}\text{Cs in soil (Bq }{\text{kg}}^{-1}\text{) = 10,000 }\times \;60\;\times \;60\;\times \;24\;\times \;365\;\times \;2.06\;\times \;2^{\text{\textasciicircum{}}}(1/2)/(6.02\;\times \;10^{23})\\ =\;1.54\text{ pmol }{\text{kg}}^{-1}\;=\;206\text{ pg }{\text{kg}}^{-1}\\ 10,000\;{\text{Bq;}}^{134}\text{Cs in soil (Bq }{\text{kg}}^{-1}\text{) = 10,000 }\times \;60\;\times \;60\;\times \;24\;\times \;365\;\times \;30.2\;\times \;2^{\text{\textasciicircum{}}}(1/2)/(6.02\;\times \;10^{23})\\ =\;23\text{ pmol }{\text{kg}}^{-1}\;=\;3.15\text{ ng }{\text{kg}}^{-1} \end{array}$$

**FIGURE 3:**
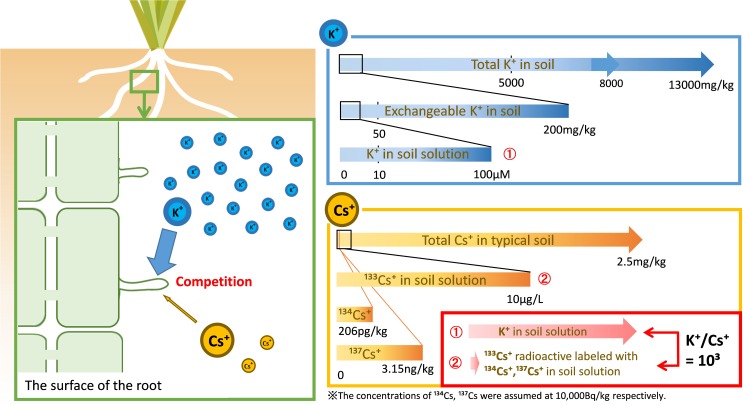
The Cs targeted in this review. The amount of stable isotope Cs (^133^Cs) in a typical soil is approximately 2.5 mg/kg but its concentration in soil solution is lower than 10 μg/L (75 nM) (Tsukada et al., 2002; Rai et al., 2017). Our target in this review is radioactively labeled ^133^Cs and a much smaller amount of ^134,137^Cs. In contrast to radio Cs^+^, potassium is an environmentally abundant element supplied from soil clay minerals, at concentrations of 5000–13,000 mg/kg soil (Tsukada et al., 2002). The exchangeable K^+^ available to plants is approximately 200 mg/kg soil, while 10–100 μM K^+^ is retained in soil solution (Sparks and Huang, 1985; Gomez-Porras et al., 2012; Kitagawa et al., 2017). The actual K/Cs ratio is approximately 10^3^ in the soil solution.

Even if the concentration of radioactive Cs in the soil is 10,000 Bq/kg, the amount of ^134^Cs and ^137^Cs is 206 pg kg^–1^ and 3.15 ng kg^–1^, respectively, which is negligible for the amount of stable isotope Cs. Cs that is the focus of this review is ^133^Cs radioactively labeled with a considerably lower concentration of ^134,137^Cs (Figure 3, bottom right panel).

It is well known that Cs^+^ competes with K^+^ during plant uptake and it is absorbed by plants through the same mechanism as K^+^. The quantitative relationship between K^+^ and Cs^+^ controls for the Cs^+^ uptake of plants (Figure 3, left panel).

K is the fourth most abundant element in the lithosphere, and its ion, K^+^, is supplied by the soil clay minerals. The total K concentration in paddy fields is approximately 8000 mg kg^–1^ (5000–13,000 mg kg^–1^) (Tsukada et al., 2002). The concentration of exchangeable K^+^ is an indicator of plant available K^+^. The exchangeable K^+^ concentration in 178 agricultural soils (95 paddy fields and 83 upland fields in Japan) ranged from 43 to 1304 mg kg^–1^, with a median of 209 mg kg^–1^ (Kitagawa et al., 2017). The concentration of K^+^ in soil solution is less than 10% of exchangeable K^+^ concentration. Furthermore, the concentration of K^+^ in soil solution is variable in the rhizosphere as the rate of exchangeable K^+^ release is slower than the rate of K^+^ uptake by plants; 10–100 μM K^+^ is contained in soil solution (Sparks and Huang, 1985; Ashley et al., 2005; Gomez-Porras et al., 2012; Figure 3, upper right panel).

As described above, K^+^ concentration in soil solutions is 10–100 μM and that of Cs^+^ is less than 100 nM. The difference between the K^+^ and Cs^+^ concentrations in soil solution is large and the K/Cs ratio is approximately 10^3^ in the soil solution (Figure 3, bottom right panel). Some studies on Cs^+^ uptake by plants for functional analysis, such as a selectivity of ions of the K^+^ transporter, were conducted at equal concentration of Cs^+^ and K^+^, which therefore differed greatly from the concentration of Cs^+^ in the environment. In the dynamics of radio-Cs between soils and plants, this difference should be considered.

## The Mechanism of K^+^ Uptake in Higher Plants

Since the time Collander (1941) proposed his theory that K^+^ and Cs^+^ are absorbed by roots through the same pathway, the research on Cs^+^ uptake has always been associated with the analysis of the K^+^ uptake mechanism. As described in section “Introduction,” K^+^ mainly regulates the osmotic pressure of plant cells, and its concentration in a cell itself affects the activity of enzymes. Plants use various K^+^ transporters on the cell membrane to adjust the intracellular K^+^ concentration depending on the plant part or tissue. Thus, the mechanism of intracellular K^+^ concentration adjustment is intricate and involves a complex K^+^ transport network in which a considerable number of K^+^ transporters continuously cooperate to provide required concentrations of K^+^ to all plant cells (Maathuis, 2009).

Genomic analyses identified 35 genes in Arabidopsis and 50 genes in rice controlling K^+^ transporters and the uptake or excretion of K^+^ across cell membranes (Amrutha et al., 2007; Yang et al., 2009). There are a large number of K^+^ transporters, divided into five major K^+^ transporter families based on their functional type (Mäser et al., 2001). To date, only a part of the K^+^ uptake and transport pathways and their many transporters have been elucidated. One of them is K^+^ uptake in roots.

The uptake of K^+^ into plant cells using K^+^ transporters is driven by the H^+^ concentration gradient and electric potential difference caused by H^+^ transport out of the cells. K^+^ transporters that take K^+^ into the cells are mainly divided into a carrier type and channel type. Carrier-type K^+^ transporters include the KUP/HAK/KT (or shorter HAK) family of high affinity K^+^ transporters, which transport K^+^ into the cells by co-transport with H^+^. The transport by HAK requires binding of K^+^ and H^+^ to the HAK protein itself; therefore, the absorption curve plotting the external K^+^ concentration and the rate of K^+^ carried into the cells fits the Michaelis–Menten equation (Maathuis and Sanders, 1994). Carrier-type K^+^ transporters have high affinity for K^+^ and can transfer K^+^ into the cells even at low extracellular K^+^ concentrations. The HAK family is the most numerous among the K^+^ transporters, and it is encoded by genes such as *AtHAK1*–*AtHAK13* in Arabidopsis and *OsHAK1*–*OsHAK27* in rice (Gupta et al., 2008; Yang et al., 2009).

In contrast, the channel type of K^+^ transporters carries K^+^ into the cells when the extracellular K^+^ concentration is relatively high. There are two types of K^+^ channels: an inward rectifying K^+^ (KIR) channel that transports K^+^ into the cells, and an outward rectifying K^+^ (KOR) channel that flows K^+^ out of the cells (Rodríguez-Navarro, 2000; Szczerba et al., 2009).

Generally, the concentration of K^+^ in plant cells is 100 mM or higher, while its concentration in soil solution is less than 1 mM even in K^+^-rich soils. Therefore, the KIR channel functions (rectifying property) only when the membrane potential becomes more negative than the equilibrium potential of K^+^ and the direction of K^+^ transmission is predetermined. Channel-type K^+^ transporters do not require the binding of K^+^ to channel proteins and can pass more than 10^6^ ions per second.

**FIGURE 4:**
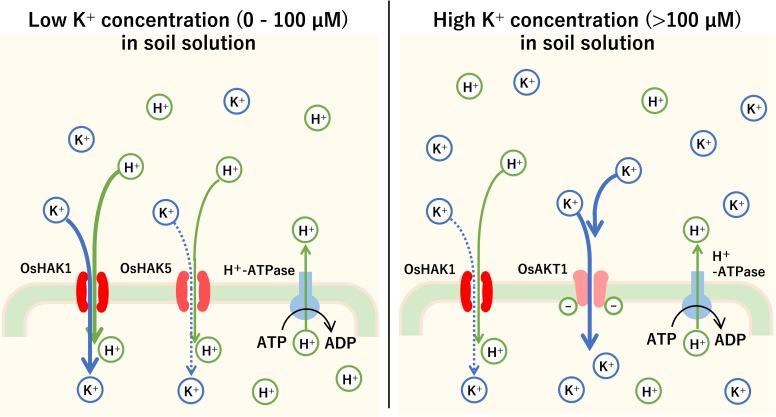
The mechanism of K^+^ uptake into the plant roots. Root cells in plants pump H^+^ out of cells successively with H^+^-ATPase. Using the H^+^ concentration gradient and the electrical potential difference across the cell membrane as driving force, the root cells absorb K^+^ by carrier or channel-type K^+^ transporters. In rice, when the K^+^ concentration is low (0–100 μM), high-affinity K^+^ (HAK) transporters such as OsHAK1 and OsHAK5 mainly transport K^+^ by symport with H^+^. Above 100 μM, KIR channels such as OsAKT1 cause an influx of K^+^ into root cells.

It has been shown that switching between the carrier type and channel type during K^+^ uptake into plant roots depends on K^+^ concentration in the soil. At low soil K^+^ concentrations (less than 1 mM), HAK family such as OsHAK1 and OsHAK5 in rice and AtHAK5 and AtKUP7 in Arabidopsis is mainly activated (Pyo et al., 2010). However, at high soil K^+^ concentrations (1 mM or more), channels such as OsAKT1 in rice and AtAKT1 in Arabidopsis, belonging to the KIR channel family, are the main transporters of K^+^ (Nieves-Cordones et al., 2016; Figure 4). For example, the *K*m of AtHAK5 was 15–24 μM K^+^, while that of AKT1 was 0.88 mM (Gierth et al., 2005).

However, of the 27 HAK transporters in rice, the expression levels of OsHAK1, 7, and 16 are always high in rice roots, whereas the other HAK transporters are highly expressed during K^+^ deficiency (Okada et al., 2008). Thus, not all routes of K^+^ uptake are unknown. After absorption into root cells, K^+^ is moved from epidermal cells into cambium via cytoplasmic communication, and then released from the adjacent vessel cells through the KOR channel.

## Cesium Permeability of Potassium Transporters

Transport of Cs^+^ by K^+^ transporters has been investigated by competition analysis in various plants by altering the ionic composition of hydroponic solutions, by electrophysiological methods using microelectrodes, and by investigating Cs^+^ uptake characteristics of knockout mutants for K^+^ transporters in model plants such as Arabidopsis. Studies employing the first two types of experiments are detailed in a review by White and Broadley (2000).

Since the time Collander (1941) proposed his theory about K^+^ and Cs^+^ uptake by roots, the uptake of these alkali metal ions was analyzed in hydroponic cultivations of barley and wheat. A study found that the rate of Cs^+^ influx under different Cs^+^ concentrations can be described by the sum of two Michaelis–Menten hyperbolae (Epstein, 1972). This suggests that Cs^+^ may be absorbed into the roots by two types of K^+^ transporters (high affinity and low affinity) having different *K*_m_. Shaw and Bell (1989) examined the concentration dependence of Cs^+^ influx into excised wheat roots and proposed the existence of two distinct Cs^+^ influx isotherms. These authors reported that at excessive Cs^+^ concentrations, the Cs^+^ influx into the roots increases linearly with increasing Cs^+^ concentration in the hydroponic solution, and they suggested that this second uptake system had lower affinity (Shaw and Bell, 1989).

The inhibition of Cs^+^ influx by other ions has also been analyzed. The inhibition increased in the following order: Li^+^ < Na^+^ < NH_4_^+^ < Rb^+^ ≤ K^+^ (Bange and Overstreet, 1960; Handley and Overstreet, 1961; Shaw and Bell, 1991). For divalent ions, the inhibition increased in the following order: Ca^2+^ < Mg^2+^ ≤ Ba^2+^ (Handley and Overstreet, 1961), although the inhibition by divalent ions was incomplete. In spinach, an increase in the Ca^2+^ and Mg^2+^ concentrations significantly decreased Cs^+^ uptake (Smolders et al., 1997).

Thus, it is possible that Cs^+^ influx is associated with several K^+^ transporters that may be inhibited by Ca^2+^ concentration. Cs is taken up by at least two types of transporters with different properties. However, it should be highlighted that in the studies utilizing hydroponics, the concentration of Cs^+^ used in the experiments was sometimes much higher than that found in soil environments under normal conditions (less than 10 ppb; 75 nM Cs^+^ in soil solution), since these studies focus on the difference in the uptake rate between K^+^ and Cs^+^.

Electrochemical studies have shown that high-affinity HAK, low-affinity KIR channels, KOR channels, and voltage-insensitive cation channels (VIC) are Cs^+^ permeable (White and Broadley, 2000). The ratio of Cs^+^ permeability to K^+^ influx of KIR channel in barley is 0.39–0.43 and much lower, at 0.07, in Arabidopsis (Wegner and Raschke, 1994; Maathuis and Sanders, 1995). Cs permeability of KIR channels is lower than that of K^+^. Furthermore, increasing Cs^+^ concentration inhibits K^+^ permeation of KIR channels (Bregante et al., 1997; White, 1997).

The genes for several KIR channels have been cloned and expressed in *Xenopus* oocytes, yeast, and other cell types to study the characteristics of KIR channels electrophysiologically (Dreyer et al., 1999). Cation influx through KIR channels is inhibited in a voltage-dependent manner by extracellular Cs^+^ (Ichida and Schroeder, 1996; Dreyer et al., 1999). Therefore, although Cs^+^ can physically diffuse through the KIR channels, practically it can hardly permeate from the soil solution in the natural environment (White and Broadley, 2000).

VIC channels have non-selective cation permeability and voltage independence (insensitivity). The permeability of Cs^+^ through the VIC channels in rye roots was 0.85 when that of K^+^ was defined as 1.0 (White and Tester, 1992). Cation influx through the VIC channels is insensitive to Cs^+^, but is partly inhibited by Ca^2+^, Ba^2+^, and other divalent ions (Roberts and Tester, 1997; White, 1997, 1999; Davenport and Tester, 2000). These characteristics correspond to the inhibition of Cs^+^ influx by divalent ions.

Finally, we introduce the results of the mutant analysis of the model plant *Arabidopsis thaliana*. In Arabidopsis, deletion mutant lines of K^+^ transporters such as HAK and AKT have been obtained to study their contribution to Cs^+^ influx.

AtHAK5 is a high-affinity K^+^ transporter that mainly absorbs K^+^ at low extracellular K^+^ concentrations. The AtHAK5 deletion significantly reduced Cs^+^ absorption, but Cs^+^ absorption was not significantly changed in AtAKT1 deletion mutants (Broadley et al., 2001; Qi et al., 2008). However, Cs^+^ uptake in *athak5* mutants during K^+^ deficiency did not decrease significantly, suggesting that Cs^+^ was absorbed by other K^+^ transporters (Qi et al., 2008). Recently, it was reported that, when grown on radioactive Cs-contaminated soil, the amount of Cs^+^ uptake by *athak5* is significantly lower than that of the wild type (Tanoi et al., 2019).

The rate of K^+^ uptake was examined in *athak5* and *atakt1* single and double mutants. At K^+^ concentrations lower than 0.01 mM, only AtHAK5 is involved in K^+^ uptake, whereas at K^+^ concentrations from 0.01 to 0.05 mM, both AtHAK5 and AtAKT1 are activated in K^+^ uptake (Rubio et al., 2008, 2010). This suggested that AtAKT1 and other low-affinity K^+^ transporters absorb K^+^ at higher K^+^ concentrations. In addition, the double *athak5* and *atakt1* mutants were able to grow at K^+^ concentrations higher than 0.05 mM, suggesting that K^+^ transporters other than AtHAK5 and AtAKT1 absorb K^+^ and complement for the lack of the two K^+^ transporters.

Caballero et al. (2012) reported that a Ca^2+^ sensitive transport system mediates low-affinity K^+^ uptake in the *atakt1* plants. The low affinity system probably belonging to the VIC channels may mediate the K^+^ uptake in *athak5*, *atakt1* plants.

In summary

I. Cs^+^ is absorbed by the same mechanism as K^+^, and their relationship is competitive.
II. Certain Cs^+^ influx pathways are also inhibited by divalent ions such as Ca^2+^.
III. Cs^+^ and K^+^ are not absorbed into the plant roots equally. It is also hypothesized that Cs^+^ inhibits the K^+^ influx through KIR channels. Cs^+^ permeability differs for HAK, KIR, and VIC channels.
IV. There are at least two Cs^+^ influx pathways into the roots (in wheat). The Cs^+^ concentration dependence of Cs^+^ influx into the roots could be drawn by the sum of two hyperbolae.
V. K^+^ uptake systems have a high level of redundancy in plants. If one of K^+^ transporters is inactivated, K^+^ influx is complemented by other K^+^ transporters (HAK, AKT, VIC). Similarly, Cs^+^ uptake system may involve multiple K^+^ transporters.

White and Broadley (2000) proposed the Cs^+^ uptake model, which was based on the characteristics of each type of K^+^ transporters (including VIC channels). The model predicted that the VIC channel and the HAK family mainly absorb Cs^+^ into the root, and KIR channels, such as AKT1, are almost unrelated to the Cs^+^ uptake (White and Broadley, 2000). However, in order to fully prove the model, it is necessary to create a plant in which each pathway has been deleted by techniques such as mutation or genome editing and examine the effect of gene deletions on Cs^+^ influx. Ultimately, the goal would be to create a plant that does not absorb Cs^+^. Unfortunately, this has not been achieved before Fukushima.

## Cs Uptake Mechanism Into Rice Roots

In the post-Fukushima studies, cultivation tests of many rice varieties in Cs-contaminated soils were conducted promptly to examine the differences in Cs uptake between varieties (Ohmori et al., 2014; Ono et al., 2014). Cs accumulation in the straw and brown rice of numerous varieties was analyzed, and the results revealed wide differences in Cs accumulation between varieties and large fluctuations in a cultivation year. These studies could not identify the causative genes for low and high Cs accumulation.

The analyses conducted in rice fields established no correlation between the concentrations of radio-Cs in soils and the amount of radio-Cs absorbed by rice plants. However, there was a correlation between the amounts of exchangeable K^+^ in the soils and the radio-Cs absorbed by rice plant (Kato et al., 2015; Kohyama et al., 2015; Kondo et al., 2015). The K^+^ levels in the soils have a greater effect on the Cs uptake of plants than the concentration of radio-Cs in the soil.

**FIGURE 5:**
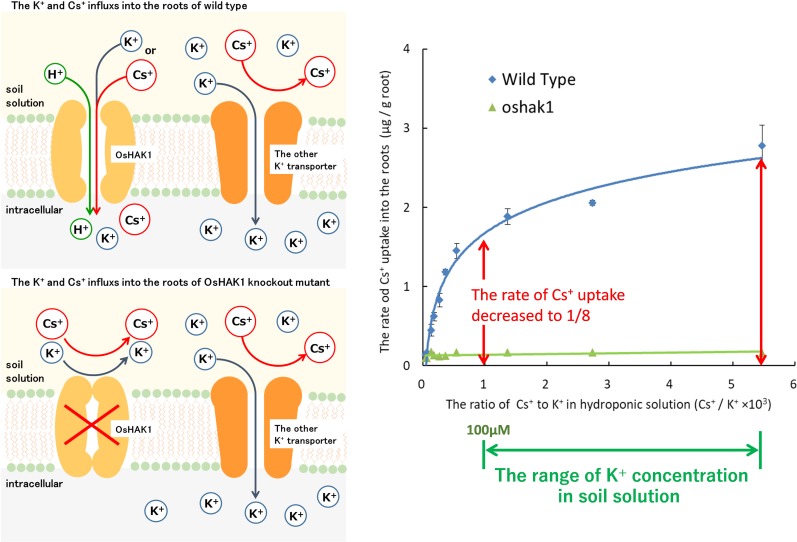
The newly elucidated mechanism of Cs uptake in the rice plants. Upper left side: The root cells of rice (wild type) absorb K^+^ and Cs^+^ through OsHAK1 at extracellular K^+^ concentration below 100 μM. Lower left side: OsHAK1 knockout mutants could not absorb K^+^ and Cs^+^ through OsHAK1. However, the rate of K^+^ uptake was maintained and the uptake was complemented by K^+^ transporters other than OsHAK1. Possibly, other K^+^ transporters other than OsHAK1 in the membrane did not permeate Cs^+^. Right figure: In typical soils, the concentration of K^+^/the concentration of Cs^+^ in soil solution is higher than 10^3^. When the K^+^ concentration in the hydroponic solution is 0–100 μM and the Cs^+^ concentration is fixed at 75 nM, the rate of Cs^+^ uptake into the root of the OsHAK1 mutant is reduced to 1/8 or less compared to the wild type. In addition, the rate of the slight Cs uptake is constant regardless of the K^+^ concentration of the hydroponic solution.

Our research group separated the mutants with low Cs^+^ uptake and identified the causative gene. Elemental analysis of brown rice in 8027 mutants, which were induced by chemical mutation treatment, isolated a mutant with low Cs^+^ uptake whose Cs concentration in brown rice was less than 10% of the wild type (Rai et al., 2017). OsHAK1, a member of the high-affinity HAK family expressed in roots, was identified as the causative gene. During hydroponic cultivation with multiple K^+^ concentrations, the Cs^+^ influx into the roots of *oshak1* was significantly reduced by less than 1/8 of the Cs^+^ influx in wild type under low to normal soil K^+^ concentrations. When this mutant was cultivated in a paddy field with high ^134,137^Cs and low K^+^ concentration, the ^137^Cs of wild-type brown rice was 44 Bq/kg, whereas that of *oshak1* was lower than the detection limit (4.92 Bq/kg). Although the absorption of Cs^+^ decreased, the absorption of K^+^ was not significantly different from that of the wild type (Rai et al., 2017; Figure 5). This indicated that other K^+^ transporters complemented the K^+^ uptake of OsHAK1. It has been also suggested that K^+^ transporters other than OsHAK1 expressed on the surface of rice roots (mainly OsAKT1) hardly absorb Cs^+^. This is in agreement with the results of Cs^+^ permeability analysis in higher plants (Wegner and Raschke, 1994; Maathuis and Sanders, 1995).

The contribution of OsHAK1 to K^+^ absorption increases under low K^+^ conditions (50–55% in the range of 50–100 μM K^+^ and 30% at 1 mM K^+^) (Chen et al., 2015). The expression of OsHAK1 is induced 8–12-fold under K^+^ deficiency, but suppressed under increased external K^+^ concentration (Okada et al., 2008; Chen et al., 2015). These findings suggest that the use of potassium fertilizer not only reduces the Cs^+^ uptake rate by increasing the K^+^:Cs^+^ ratio, but also reduces the Cs^+^ uptake by suppressing OsHAK1 expression.

The contribution of OsHAK1 for Cs^+^ uptake has been also analyzed by reverse genetic techniques. The knockout lines that have lost the function of OsHAK1 created by CRISPR-Cas9 genome editing did not depolarize even when the roots were immersed in Cs^+^ solutions with different concentrations (Nieves-Cordones et al., 2017). This indicated that Cs^+^ was not absorbed into the root cells and the root membrane potential remained unaffected. It has also been reported that, when cultivated on K^+^-deficient soil contaminated with radio-Cs, the amount of ^134,137^Cs absorbed was significantly lower than that of the wild type (Nieves-Cordones et al., 2017).

Both the forward and reverse gene techniques identified the same causative gene, revealing that most of the Cs^+^ uptake in rice plants takes place via OsHAK1. Cs absorption could be reduced by indirectly suppressing HAK expression (Ishikawa et al., 2017). From the mutants irradiated with accelerated carbon ions, a low-Cs mutant was obtained that could reduce the radio-Cs uptake to about 30% of that of the wild type. The causative gene in this mutant was *OsSOS2*. This gene encodes a kinase that phosphorylates OsSOS1 (Na^+^/H^+^ antiporter) in rice root cells. Considering that OsSOS1 is activated by phosphorylation, knockout of *OsSOS2* inactivates OsSOS1 and reduces the Na^+^ extrusion from roots. Increased intracellular Na^+^ concentration is sensed by the cell as an increase in osmotic pressure, which triggers the cell to suppresses the expression of K^+^ transporters such as *HAK* (*OsHAK1*, *OsHAK5*), *OsAKT1*, and *OsHKT2*. It ultimately results in a decreased Cs^+^ influx through these K^+^ transporters. It has been shown that the stress caused by increasing Na^+^ concentration in the root cells can also reduce Cs uptake in rice (Ishikawa et al., 2017). Thus, the mechanism of salt tolerance in rice can also be used to suppress Cs^+^ uptake.

## Cs Absorption in Other Higher Plants

Most of the radio-Cs that had fallen to land after the Fukushima nuclear accident entered the forest ecosystem. Therefore, it is also important to examine the absorption of radio-Cs in trees. An interesting dynamics of Cs^+^ transport was revealed in the model forest tree poplar. The poplar does not change its K^+^ absorption during both long-day period and short-day period. However, the rate of Cs^+^ uptake by poplar treated for 6 weeks on short days decreased to about 1/4 of that in poplar treated for 9 days on a long day. Furthermore, the expression levels of major HAK and VIC channels did not change, suggesting that the tree may have an unknown Cs^+^ absorption pathway (Noda et al., 2016).

After absorption into root cells, K^+^ and Cs^+^ are moved to cambium via cytoplasmic communication, and then released from the adjacent vessel cells via the KOR channels. One of the KOR channels, stelar K^+^ outward rectifying channel (SKOR), has been identified in Arabidopsis. The SKOR is involved in the permeation of K^+^, Rb^+^, and Cs^+^. However, as the results were obtained by heterologous expression, the pathway of Cs^+^ in the plant body could not be elucidated (Gaymard et al., 1998; Johansson et al., 2006).

The molecular recognition of Cs^+^ and K^+^ in the K^+^ transporter has also been reported. Mutations were randomly introduced by PCR in cDNA synthesized from AtHAK5 of *A. thaliana* and heterologously expressed in yeast. When amino acid substitution occurred between the second and third transmembrane regions containing the K^+^ binding hole, the selectivity of K^+^ was 100-fold higher than that for Na^+^ or Cs^+^ (Alemán et al., 2014). This points to a possibility that the selectivity of the high-affinity K^+^ transporter for alkali metal ions can be further improved, and that only K^+^ is incorporated into the cell by editing the amino acid sequence of HAK by mutagenesis. It may be possible to produce plants with K^+^ transporters that are highly selective toward K^+^ absorption (as well as plants that preferentially accumulate Cs^+^). The plant K^+^ transporter may have undergone evolutionary pressure for selective permeability of alkali metal ions.

## Comparing the Pathway of Cs^+^ Uptake in Rice Roots With the Model Proposed by White and Broadley

White and Broadley (2000) proposed a model for Cs^+^ uptake, in which VIC channels contribute most of the Cs^+^ influx under realistic soil conditions and HAK transporters carry the residual Cs^+^ influx. In this section, we verify whether the Cs^+^ and K^+^ uptake in rice fits this model, which involves HAK, KIR channels, and VIC channels.

### Function of HAK in Cs^+^ and K^+^ Uptake by the Roots of Rice Plants

In rice grown in a hydroponic solution with normal soil K^+^ concentration (0–100 μM), OsHAK1 absorbed most Cs^+^ into the roots. When K^+^ concentration in the hydroponic solution increased, the amount of Cs^+^ uptake into the roots decreased drastically due to competition with K^+^. At K^+^ concentrations above 1 mM, there was no difference in Cs^+^ uptake between wild-type and *OsHAK1* mutant (Rai et al., 2017). When the K^+^ concentration was increased from 14 μM to 1.1 mM, the Cs^+^ uptake was reduced from 2.8 to 0.13 μg g^–1^ roots, and it did not decrease further (Figure 5 right panel). It is assumed that even at high K^+^ concentrations, a small amount of Cs^+^ is carried into the roots by transporters other than the K^+^ transporter. The predicted mechanism of Cs^+^ uptake under high K^+^ condition is described in section “Function of VIC Channels in Cs^+^ Uptake by the Roots of Rice Plants” (Rai et al., 2017).

In the simulation by White and Broadley (2000), at K^+^ concentrations between 10 μM and 1 mM, the Cs^+^ influx (Cs^+^ concentration: 0.1 μM) is reduced to only about 1/4. Bossemeyer et al. (1989) reported that the KUP transporter in *Escherichia coli* has a *K*_m_ of 5 mM for Cs^+^ transport and a *K*_m_ of 0.37 mM for K^+^ transport. The simulation was based on the fact that *K*_m_ of HAK for Cs^+^ transport is 10-fold higher than the *K*_m_ for K^+^ transport.

However, Bañuelos et al. (2002) analyzed the Cs^+^ transport functions of OsHAK1 in heterologous expression and revealed that the yeast-expressed chimeric OsHAK1 (a part of the barley HvHAK1 sequence was added to the incomplete OsHAK1 cDNA) can transport Cs^+^ and K^+^ with a *K*_m_ of 11 and 6 μM, respectively. Thus, the difference in *K*_m_ for Cs^+^ and K^+^ transport may be smaller (Cs: up to 2-fold, K^+^: up to 11-fold) compared with the difference predicted by their model (White and Broadley, 2000; Bañuelos et al., 2002).

OsHAK1 is a high-affinity transporter that contributes to K^+^ uptake mainly under low K^+^ concentration, but under extracellular K^+^ deficiency, the expression levels of this transporter in the roots increase several-fold (Okada et al., 2008, 2018; Nieves-Cordones et al., 2016). Thus, the responses of rice plants indicate that the contribution of HAK (OsHAK1) to Cs^+^ uptake may be higher than predicted by their model.

### Function of KIR Channels (OsAKT1) in Cs^+^ and K^+^ Uptake by the Roots of Rice Plants

Deletion of *OsHAK1* drastically reduces Cs^+^ uptake, but the K^+^ uptake rates do not differ greatly between the OsHAK1 mutant and the wild type. This was attributed to other systems, which are involved in K^+^ absorption into the roots, but contributed much less to Cs^+^ uptake in OsHAK1 mutants. In rice roots, OsHAK1 and OsAKT1 mediate the K^+^ uptake according to the extracellular K^+^ concentration (Nieves-Cordones et al., 2016). In *oshak1*, probably most of K^+^ that is typically absorbed by OsHAK1 is transported by OsAKT1. Although the KIR channel is a low affinity K^+^ uptake system, the overexpression of OsAKT1 could improve the K^+^ uptake under 100 μM K^+^ conditions. OsAKT1-mediated K^+^ flux may be small but can generate a sizeable contribution over prolonged periods (Ahmad et al., 2016).

The current of KAT1 in Arabidopsis, a kind of KIR, is blocked by Cs^+^; it was reduced by 20% at Cs^+^ concentration of 0.1 mM, and by 50% at 0.5 mM Cs^+^ (Ichida and Schroeder, 1996). However, because the concentration of ^133^Cs in the soil solution (below Cs^+^: 10 ppb,75 nM) is considerably lower than the concentration of Cs^+^ examined in these studies, it is likely that OsAKT1 is not blocked by Cs^+^ and thus can complement for the loss of K^+^ absorption caused by the absence of OsHAK1. In addition, as we discuss below, it is almost certain that OsAKT1 does not contribute to Cs^+^ influx because the remaining Cs^+^ uptake is not affected by K^+^.

### Function of VIC Channels in Cs^+^ Uptake by the Roots of Rice Plants

Even with OsHAK1 knockout, a small amount of Cs^+^ was incorporated into rice roots. The residual Cs^+^ uptake in OsHAK1 mutant was not affected by the extracellular K^+^ concentration. The model proposed by White and Broadley (2000) showed that a high amount of Cs^+^ entered into the roots through the VIC channels. A relative permeability sequence of K^+^:Rb^+^:Cs^+^:Na^+^:Li^+^ of 1.00:1.00:0.85:0.73:0.71 (K^+^ = Rb^+^ > Cs^+^ > Na^+^ > Li^+^) was given by the electrical conductance measurements of the VIC channels on the plasma membrane in the rye roots (White and Tester, 1992). In wheat roots, the selectivity relative to Na^+^ was NH_4_^+^ (2.06) > Rb^+^ (1.38) > K^+^ (1.23) = Cs^+^ (1.18) > Li^+^ (0.83) > TEA^+^ (0.21) = Ca^2+^ (0.21) (Davenport and Tester, 2000). On the basis of the results, Cs^+^ permeability via the VIC channels is much higher than that via KIR channels. Cation influx through the VIC channels is partly inhibited by Ca^2+^, Ba^2+^, and other divalent ions (Roberts and Tester, 1997; White, 1997, 1999; Davenport and Tester, 2000).

These characteristics of VIC channels correspond to the inhibition of Cs^+^ influx of the plants by divalent ions (Handley and Overstreet, 1961; Smolders et al., 1997). Therefore, the uptake of residual Cs^+^ in rice roots may have occurred through the VIC channels.

However, the contribution of the VIC channels in rice roots was considerably lower than that of OsHAK1, which was the opposite of what was predicted by their model. A possible reason is that the Cs^+^ concentration in soil solution was lower than that assumed in their model. Given that the amount of Cs^+^ uptake varies greatly depending on the extracellular K^+^ concentration, the contribution of each transporter to Cs^+^ uptake depends greatly on the value of extracellular K^+^ concentration that is assumed standard. For example, if K^+^ concentration is high, the contribution of HAK is lower and the contribution of other transporters (such as VIC channels) becomes high. Therefore, in this review, we discussed the contribution of each transporter to Cs^+^ uptake under K^+^ and Cs^+^ concentrations typically found in actual soils.

The other reason for the discrepancy between the model and our results is that rice plants have remarkably high silica content. Rice plants actively absorb silicic acid, and its content in the stem and leaves ranges from 10 to 20%. In contrast, Ca^2+^ content of rice plants (0.3 to 0.4%, w/w) is lower than that recorded in other plants (average of 1.57%). Therefore, it was suggested that the Ca^2+^ concentration in the rhizosphere or apoplast of rice plants is higher than that in other plants because rice plants do not absorb high amounts of Ca^2+^. Future research should analyze which VIC channels are involved in Cs^+^ uptake into the roots by means of molecular techniques, mutations, or other methods.

**FIGURE 6:**
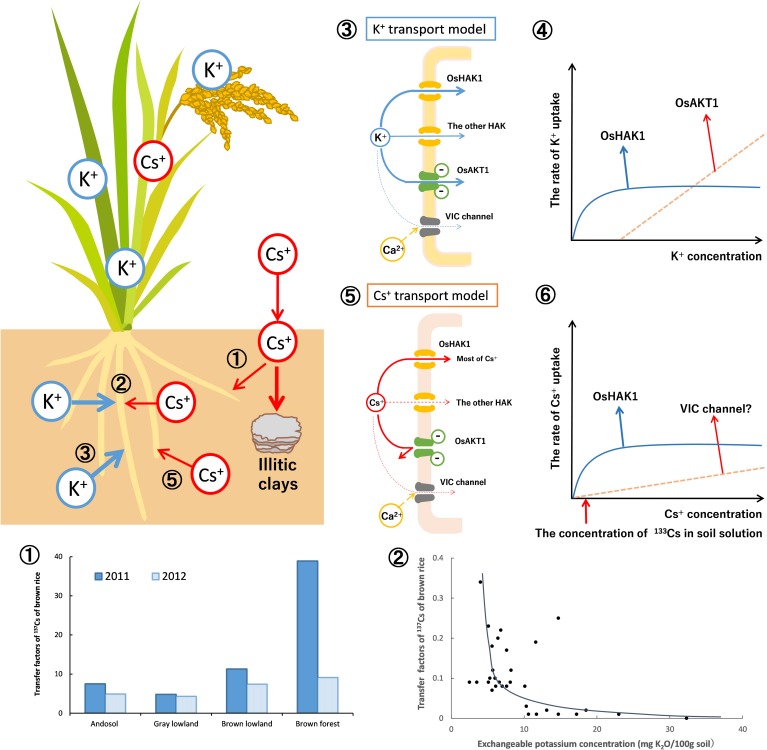
Dynamics of radioactive Cs between soil and plant (using rice as an example). The dynamics of Cs^+^ in soils was mainly affected by the specific adsorption to soil clay material (frayed edge sites of mica) and the competition with K^+^ in the uptake by plants. As described in Figure 2, the newly entered Cs^+^ (radio Cs^+^) would be eventually immobilized. Therefore, available Cs^+^ and its transfer factors in the rice grains decreased yearly (➀). The graph is drawn from the data of Fujimura et al. (2015). The relationship between the soils-to-rice-plant transfer factors and the concentration of exchangeable potassium in soils was shown (➁). The K^+^ and Cs^+^ compete for absorption by the rice roots. While exchangeable (plant-available) K^+^ in soils increased, the Cs^+^ concentrations in rice grains decreased drastically. The graph is drawn from the data of Kohyama et al. (2015). The K^+^ and Cs^+^ uptake in rice plants by the membrane transporters is estimated. In ➂, the mode of K^+^ uptake in the roots of rice plants is proposed. K^+^ uptake is mainly through OsHAK1 and OsAKT1. In ➃, the K^+^ uptake curve is composed of two distinct transporter systems as shown by Epstein (1972). The solid line indicates the rate of K^+^ uptake by OsHAK1 in rice while the dotted line indicates the K^+^ influx through OsAKT1 channel. The rest of K^+^ uptake is contributed by the other K^+^ transporters. In ➄, the mode of Cs^+^ uptake is proposed. Cs^+^ uptake is through OsHAK1 and the VIC channel. In ➅, the Cs^+^ uptake curve also consists of two Michaelis–Menten hyperbolae as shown by Shaw and Bell (1989). The solid line indicates the rate of Cs^+^ uptake by OsHAK1 in rice and the dotted line indicates potential Cs^+^ influx through the VIC channel. Since the Cs^+^ concentration in typical soil solution is lower than 75 nM (10 ppb:^133^Cs, as the red arrow indicates), it is estimated that most of the Cs^+^ uptake in rice is absorbed by OsHAK1 and the rest through the VIC channel as the latter does not compete with K^+^.

Thus, the results of rice analysis indicated that, under normal soil conditions, OsHAK1 mediate 80–90% of Cs^+^ uptake into the roots and the rest is mediated by VIC channels (Rai et al., 2017; Figure 6-➄➅). The proportion each transporter contributed to the Cs uptake was different, but they mostly agreed with the prediction by White and Broadley (2000).

## Summary of the Dynamics of Radio-Cs From Soil to Plants

The key point in the dynamics of radio-Cs in soils is its immobilization in soils. The amount of frayed edge sites in soils have significant effects on the radio-Cs uptake by plants. The free radio-Cs strongly binds to frayed edge sites in the soil, which decreases available radio-Cs for plants with time. This phenomenon is important for predicting the radio-Cs uptake of plants in addition to the decay of radioactivity of ^134,137^Cs in soils (Figure 6 ➀).

In the dynamics of radio-Cs from soils to plants, the concentration of K^+^ in soil solution affects the radio-Cs uptake into plants because K^+^ and Cs^+^ compete for absorption into plant roots. While the available K^+^ concentration in soils increases, the Cs^+^ uptake by plants decreases drastically (Zhu and Smolders, 2000; Kato et al., 2015; Kohyama et al., 2015; Kondo et al., 2015). Therefore, the application of potassium fertilizers is effective for preventing the agricultural crops from absorbing radio-Cs in contaminated fields (Figure 6 ➁). Increasing the K^+^ concentration in the rhizosphere is also important because it regulates the expression of HAK, which absorb Cs^+^ into the roots.

In rice roots, OsHAK1 and OsAKT1 mediate the K^+^ uptake according to the extracellular K^+^ concentration (Nieves-Cordones et al., 2016; Figure 6 ➂). The K^+^ uptake curve is divided into the two hyperbolae (Epstein, 1972). The K^+^ uptake under low K^+^ concentration depends upon OsHAK1, and the K^+^ influx under high K^+^ levels is mediated mainly by the OsAKT1 channel (Figure 6 ➃).

The Cs^+^ uptake curve in wheat at 200 μM Cs^+^ could also be described by the sum of two Michaelis–Menten hyperbolae (Shaw and Bell, 1989). It is estimated that most of the Cs^+^ uptake in rice is via OsHAK1 under normal soil K^+^ concentrations. Permeability of KIR channels to Cs^+^ is lower than that to K^+^; practically, Cs^+^ can hardly permeate. The residual Cs^+^ influx into roots does not compete with K^+^, suggesting that it may be via transporters not involved in K^+^ uptake (Figure 6 ➄). The uptake of low affinity Cs^+^ is inhibited by divalent cations (Handley and Overstreet, 1961), whereas the permeability of monovalent cations through VIC channel is partly inhibited by Ca^2+^ (Davenport and Tester, 2000). It is reasonable to conclude that the residual Cs^+^ influx is through VIC channels (Figure 6 ➅).

## Conclusion

Humans have experienced extensive radionuclide contamination from atmospheric nuclear tests and the two nuclear power plant accidents. Nuclear tests in the atmosphere have already been prohibited, but the nuclear power accounts for 10% of global electricity production. Therefore, the risk of nuclear incidents must be considered and we must cope with the risks. Radio-Cs (^134,137^Cs) described in this review, is produced in large amounts in the reactor core. It has a low boiling point and easily diffuses into the environment after accidents. Furthermore, it has a long half-life and strongly adsorbs to soil, remaining in the environment for a long time. In unforeseen circumstances, radio Cs can be absorbed by plants and enter the food chain.

Since Fukushima, numerous studies have been conducted in Japan, including those on radio-Cs dynamics in the environment, radionuclide testing of agricultural products, and the cultivation of various crops. In addition, our understanding of the mechanism of Cs^+^ uptake in rice has rapidly progressed owing to the implementation of genetic and molecular techniques such as mutations and genome editing. As shown in this review, many researchers have conducted hydroponics experiments with various ion conditions, heterologous expression analysis of K^+^ transporters using *Xenopus oocyte* and yeast, electrophysiological analysis using microelectrodes, and analysis of mutants by forward and reverse genetics. Thus, different pathways were implemented to elucidate the entire mechanism of Cs^+^ (K^+^) uptake and transport. These experimental results are expected to reveal the Cs^+^ dynamics in soil, from soil to plant, and in individual plants. They will also lead to deeper understanding of the risks of nuclear power plants (problems caused by radio-Cs) to soil–plant ecosystems.

## Author Contributions

HR and MK made substantial direct and intellectual contribution to this work and approved the manuscript for publication.

## Conflict of Interest

The authors declare that the research was conducted in the absence of any commercial or financial relationships that could be construed as a potential conflict of interest.
